# Childhood adversity, emergent psychopathology, and adolescent-to-parent violence: Process mining trajectories from police and health service administrative data

**DOI:** 10.3389/frcha.2023.1074861

**Published:** 2023-04-11

**Authors:** Allison Peck, Marie Hutchinson, Steve Provost

**Affiliations:** Faculty of Health, Southern Cross University, Coffs Harbour, NSW, Australia

**Keywords:** child and adolescent psychiatry, anxiety disorders, depression and mood disorders, process mining, statistics and research methods, adolescent-to-parent violence

## Abstract

**Aim:**

To discover developmental risk trajectories for emerging mental health problems among a sample of adolescent family violence offenders to inform service delivery focused on early preventative interventions with children and their families.

**Design:**

A retrospective case-series design employing data linkage.

**Setting:**

An Australian regional location.

**Participants:**

Adolescents (born between 1994 and 2006) issued a legal action by the NSW Police Force for an adolescent-to-parent family violence offense (*n* = 775).

**Procedure:**

Discrete routinely collected episode data in police and health service electronic records for children, and police data for parents, were linked and transformed into longitudinal person-based records from birth to 19 years to identify trajectories for mental health problems.

**Results:**

Sixty-three percent (*n* = 489) of adolescents had contact with a mental health service before age 19. The majority of these adolescents received a diagnosis for a stress or anxiety disorder (*n* = 200). Trajectory analysis found childhood exposure to parental intimate partner violence and parental drug and/or alcohol use were dominant events in the pathway to receiving a mental health diagnosis. Being a victim of a sexual offense was found to increase the odds of adolescents having a diagnosis for each of the main mental health categories (with the exception of drug or alcohol disorders).

**Conclusions:**

Pathways to mental health problems were characterized by inter-related adverse childhood events and poly-victimization for many adolescents. Early identification of at-risk children must be a continued focus of child health services in order to reduce and identify early emerging mental health problems.

## 1. Introduction

For young people worldwide, poor mental health represents one of the largest causes of illness and disability. Half of all mental health disorders begin to emerge by 14 years and are usually preceded by earlier non-specific psychological and behavioral difficulties (1). Systematic review evidence (2) and latent class analysis (3, 4) confirm that childhood poly-victimization is a substantial risk factor for poorer mental health outcomes and adolescent offending (5). Young people involved in the juvenile justice system have typically experienced trauma, often including neglect or abuse within the family, sexual abuse, and non-family violence related assault (6–8).

Adolescent-to-parent violence is a recognized subtype of family violence. It is defined as a pattern of behavior intended to threaten, exert power or control, or cause damage and harm to a parent (9–11). Evidence suggests that the psychological profiles of adolescents involved in violence towards a parent differs from other juvenile offenders and non-offenders (12–14). Adolescents involved in adolescent-to-parent violence are reported to have higher rates of clinical diagnoses (12), including depressive symptoms and hyperactivity (13), psychiatric hospitalizations, be medicated, and have attempted suicide (14) than adolescents involved in non-family violence related offending. However, comparatively little is known about the nexus between emerging mental health problems and juvenile offending or the epidemiology of events and pathways in the emergence of adolescent-to-parent violence.

Research employing longitudinal and cross-sectional designs has reported associations between early life trauma, chronic health problems, and mental health disorders (15–21). Exposure to four different types of adverse childhood events (ACE) is strongly associated with problematic poor mental health, drug use, and self-directed violence (22). When examining adulthood mental health outcomes, the complexity of psychiatric symptoms has been reported as greater among adults reporting child abuse (23). Establishing the timing of these events in the developmental trajectory of children is important. Growing evidence from neuroscience suggests it is more difficult to achieve positive outcomes for children who experience prolonged, severe, or frequent exposure to adverse events beyond early childhood (20). From a brain development perspective, the age at which children are exposed to violence is important to identify, as earlier intervention may mitigate harm (24). Adverse exposure during critical developmental periods is more likely to be permanent (25), and these effects are thought to endure for several generations (26).

There is an established tradition among longitudinal studies, such as the Longitudinal Study of Australian Children and the Avon Longitudinal Study of Parents and Children (27), to employ parental-report of children's mental health and child temperament (28), with other studies relying on adult-victim recall of childhood abuse and neglect (23, 29–32). Both self-report and parental-report methods are limited. Research has shown that adolescents' representations of their mental health may not be reliable (33). Moreover, adolescents involved in delinquency have been found to minimize their self-report of mental illness symptoms (34, 35). Both self-report and parental self-report methods may also be subject to bias associated with retrospective recall and social desirability. Even though research has demonstrated that reports of child abuse by people with mental health disorders can be reasonably reliable over time (36), the accuracy of this information may limit the robust temporal analysis of developmental pathways.

Electronic administrative data offers researchers access to longitudinal population-based individual level routinely collected government agency data that is free from the bias associated with retrospective recall (37, 38). In addition, the utility of this data is improved when linked across multiple agencies (37, 38). Even though a recent increase has been seen in the number of multidisciplinary studies adopting this approach to explore childhood trajectories for juvenile offenders, much of this work to date has involved the use of child protective services and juvenile justice data (39, 40).

This study aimed to discover developmental risk trajectories for emerging mental health problems among a sample of adolescent family violence offenders to inform service delivery focused on early preventative interventions with children and their families.

## 2. Methods

### 2.1. Design

A retrospective case-series design employing data linkage.

### 2.2. Participants

A case series of adolescents born between 1994 and 2006 and their parents. These adolescents were issued a legal action by the New South Wales Police Force (NSWPF, between 2008 and 2018) for committing an adolescent-to-parent family violence offense when aged between 11 and 18 years. A legal action included a NSWPF issued court attendance notice, criminal infringement notice, other infringement notice and/or cannabis or other drug caution. Under the Young Offenders Act 1997 a juvenile offender can also be issued with legal actions, including a warning, a caution, or be referred to a youth justice conference (41). An adolescent-to-parent violence-related offense included family violence-related assault, malicious damage, offense against another person or sexual assault offenses. All adolescents in the case series resided in a regional geographic locale in New South Wales, Australia. This geographic area has a population of approximately 550,000 residents and incorporates seven major regional coastal and rural townships, with residents from diverse socio-economic backgrounds.

### 2.3. Data sources

For each adolescent case from birth (age one for adolescents born in 1994) to before age 19, child and parent event data was sourced from the NSWPF Computerized Operational Policing System (COPS; data range 1995–2018). The NSWPF COPS database stores all information recorded by employees of the NSWPF relating to everyday policing activities. All incidents and information reported to or detected by police are entered into the database using structured and free-text formats. Information entered into the structured fields includes the incident category, incident start date and time, location, details of the people involved [classified by their type of involvement, e.g., person of interest (POI), witness, victim, child-at-risk], and other associated factors (i.e., whether the incident was domestic violence or alcohol related)(42).

Ambulatory outpatient and hospital presentation and admission data were sourced from three NSW Ministry of Health Data Collections: (1) the Admitted Patients Data Collection (APDC; data availability 2001–2018), (2) the Emergency Department Data Collection (EDDC; data availability 2005–2018), and (3) the Mental Health Ambulatory Data Collection (MH-AMB; data availability 2006–2018). Due to limited data availability across the NSW Ministry of Health data collections, only a small number of cases (*n* = 33) had records from birth to age five years. Therefore, health data from birth to age five was excluded from this study to protect the privacy of these cases and maintain consistency in the findings reported. For each completed episode in the EDDC, a primary diagnosis was recorded using the International Statistical Classification of Diseases and Related Health Problems, Tenth Revision, Australian Modification (ICD-10-AM), ICD-9-CM (Clinical Modification) or Systematized Nomenclature of Medicine—Clinical Terms (SNOMED-CT). Hospital admissions, including a primary diagnosis and up to 50 secondary diagnoses, were recorded using the ICD-10-AM, and mental health outpatient primary and secondary diagnoses were recorded using ICD-10-V11 (43). Australian public hospital data covers almost the entire population, particularly during childhood. These datasets provide longitudinal data on all hospital admissions, presentations and mental health service outpatient visits.

Record linkage between the NSWPF and NSW Ministry of Health data was undertaken by the Centre for Health Record Linkage (CHeReL). The center is a dedicated data linkage unit managed by the NSW Ministry of Health. In sourcing and linking identifiable data across government agencies, the CHeReL provides an ethically and legally approved process that aims to protect individual's privacy and the validity of the data provided (44). The center employs an automated blocking algorithm and machine learning to apply a linkage weight to pairs of records linked across databases available in the CHeReL's master key linkage (44). The CHeReL reports that, on average, five in 1,000 records are incorrectly matched using this probabilistic method of data linkage (44).

Before linkage was conducted, data custodian approval was granted by the NSWPF and NSW Ministry of Health. Data use was also approved by the Southern Cross University Human Research Ethics Committee (ECN-18-198) and the NSW Population and Health Services Research Ethics Committee (PHSREC 2019/ETH00173) in accordance with health research confidentially and data linkage guidelines.

### 2.4. Event and diagnostic variables

Data relating to 27 event and diagnostic variables was extracted from the linked dataset (see Tables 1, 2). The ICD-10 diagnostic codes used in the Australian Institute of Health and Welfare's definition of a mental-health related hospital separation were used to identify mental health related diagnostic events (45). Diagnostic codes were grouped into eight variables according to the DSM-5 classification of mental disorders (46). Variables were created for: neurodevelopmental disorders (F70, F79-F84, F88-F95, F98.0), psychotic disorders (F20-F25, F28, F29), mood disorders (F30, F31.0, F31.1-F31.9, F32-F34, F38, F39), stress and anxiety disorders (F40, F41, F43.0, F43.1, F43.20-F43.22, F43.28, F43.8, F43.9, F48), impulse control disorders (F63, F90-F92), personality disorders (F60-F63, F68, F69), drug or alcohol related mental disorders (F10-F15, F19), and eating disorders (F50). An “attempt suicide/self-harm” variable included receiving a NSW Health diagnosis for attempt suicide, self-harming behaviors, and suicidal ideation (X70.0-X84, Y87, Z91.5) and being recorded in an attempt suicide incident by the NSWPF. Diagnostic categories related to having contact with a mental health outpatient service where a principal diagnosis of “mental health diagnosis yet to be allocated” was recorded formed the “mental health diagnosis to be allocated” variable.

**Table 1:** Number and proportion of adolescents by mental health category, age at first diagnosis and number of NSW Health mental health related contact*s.*

Mental health related variables	Total Adolescents	Age at first diagnosis	Number of mental health related contacts
	*n* (%)	M _years_ (SD)	M (SD)
Mental health diagnosis to be allocated	436 (56)	14.3 (2.34)	20 (27.18)
Stress or anxiety disorders	200 (26)	15.0 (2.12)	19 (35.12)
Attempt suicide/self-harm	148 (19)	15.5 (1.89)	8.5 (21.38)
Drug and alcohol related mental disorders	129 (17)	16.3 (1.78)	18 (62.06)
Mood disorders	123 (16)	15.5 (1.79)	30 (65.74)
Neurodevelopmental disorders	99 (13)	13.8 (2.90)	39 (75.03)
Impulse control disorders	90 (12)	13.7 (2.86)	13 (25.23)
Personality disorders	80 (10)	14.9 (2.66)	3 (5.07)
Psychotic disorders	21 (3)	16.1 (2.16)	34 (40.18)

The eating disorders variable was removed due to low numbers <10. % is the percentage of all adolescents in the case series (*n* = 775).

**Table 2:** Number and proportion of adolescents involved in each event variable by mental health diagnosis category*.*

	Stress or anxiety disorders		Attempt suicide /self-harm		Drug and alcohol related mental disorders		Mood disorders		Neuro- developmental disorders		Impulse control disorders	
	(*n* = 200)		(*n* = 148)		(*n* = 129)		(*n* = 123)		(*n* = 99)		(*n* = 90)	
Event Variables	*n*	%	*n*	%	*n*	%	*n*	%	*n*	%	*n*	%
Adverse childhood experience												
NSWPF reported *P*-IPV exposure prior to 13 years	146	73%	102	69%	96	74%	84	68%	75	76%	66	73%
Parental drug and/or alcohol use	115	58%	84	57%	85	66%	68	55%	63	64%	57	63%
Neglect	103	52%	73	49%	74	57%	62	50%	54	55%	47	52%
Physical abuse child-at-risk (CAR)	42	21%	33	22%	22	17%	25	20%	24	24%	15	17%
Family violence assault victim	66	33%	50	34%	47	36%	31	25%	31	31%	27	30%
Family violence verbal argument victim	109	55%	74	50%	61	47%	55	45%	43	43%	45	50%
Non-family violence assault victim	115	58%	75	51%	66	51%	57	46%	52	53%	46	51%
Health related												
Sexual offense victim	64	32%	51	34%	26	20%	32	26%	33	33%	26	29%
Head trauma	59	30%	36	24%	42	33%	32	26%	28	28%	29	32%
Adverse police involvement												
Alcohol use	136	68%	93	63%	-	-	74	60%	64	65%	59	66%
Drug use	135	68%	100	68%	-	-	86	70%	66	67%	61	68%
Property offense	107	54%	74	50%	78	60%	53	43%	65	66%	48	53%
Person search/move on	152	76%	105	71%	111	86%	88	72%	73	74%	67	74%
Family violence verbal argument person of interest	160	80%	110	74%	100	78%	89	72%	75	76%	65	72%
Violent offense	90	45%	66	45%	71	55%	48	39%	55	56%	52	58%
Apprehended violence order (AVO)	192	96%	145	98%	126	98%	117	95%	93	94%	87	97%
Breach AVO	91	46%	67	45%	65	50%	54	44%	56	57%	47	52%

### 2.5. Statistical analysis

A time-stamped event log containing demographic data (project person number, gender, statistical area address) and event and diagnostic data (for each event variable in Tables 1, 2) was created from the linked data files for each case using Microsoft Excel [Version 1808; see (47) for further details on how this data was recorded in the event log and how to interpret the process models]. The combined event log for all adolescents in the case series consisted of 66,085 events. The created event log was imported into the process mining software, Disco (48).

Discovery process mining employs data mining and machine learning for pattern recognition. Disco (48) uses the fuzzy miner algorithm to reveal trajectories or processes in the event log (49). The fuzzy miner algorithm uses significance and correlation metrics to calculate which activities and paths are included within each process model. The significance metric selects activities and paths between activities undertaken by a greater number of adolescents, and the correlation metric selects only pathways between closely connected activities to include in the process model (50).

**Figure 1:**
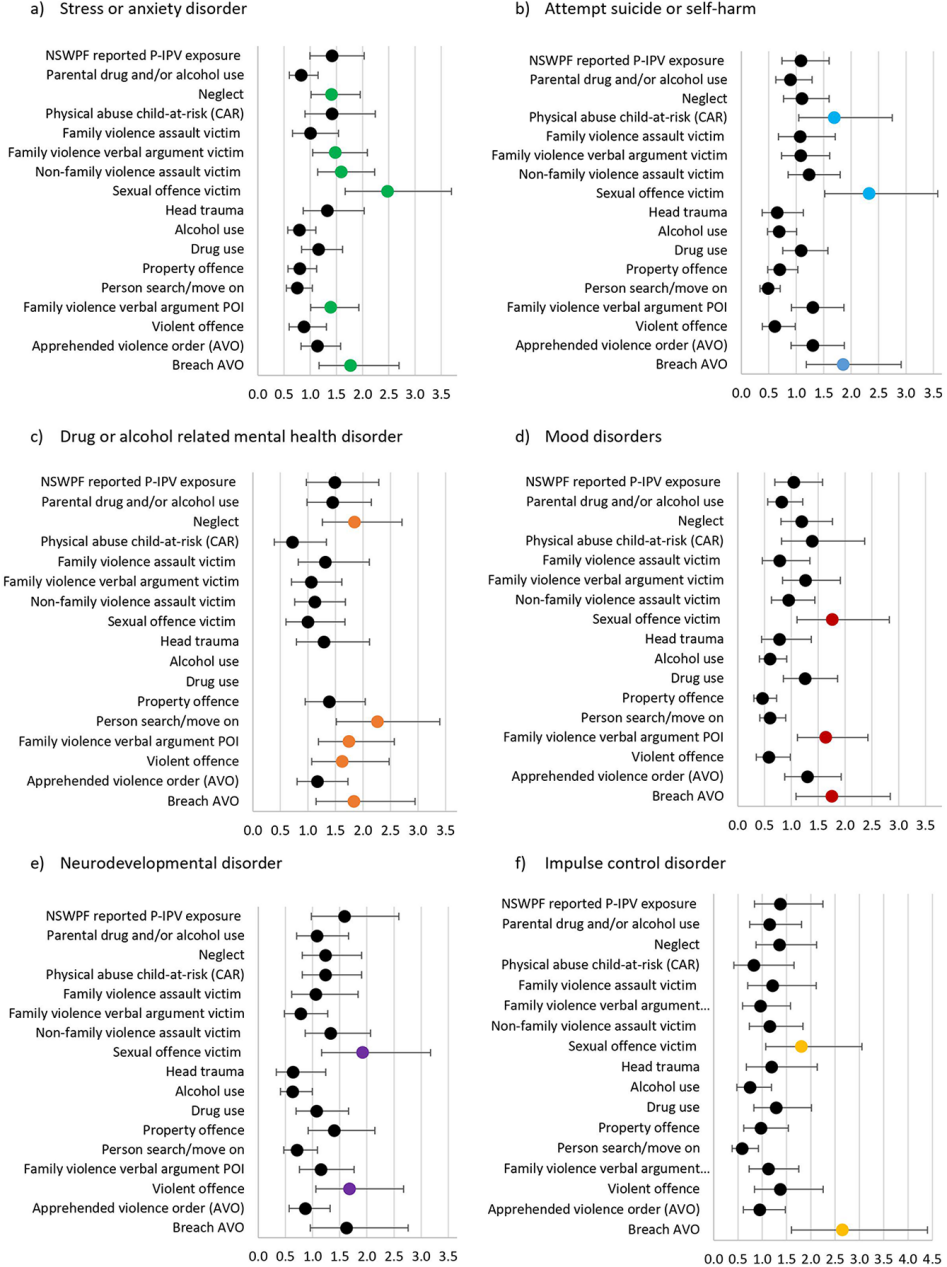
Odds ratios and 95% confidence intervals for each event variable by mental health category. (**A**) Stress or anxiety disorder (**B**) Attempt suicide or self-harm (**C**) Drug or alcohol related mental health disorder (**D**) Mood disorder (**E**) Neurodevelopmental disorder (**F**) Impulse disorder.

The process models created were imported into the ProM 6.9 process mining software (51) to assess the quality of the models. Fitness metrics, on a scale of 0 to 1, with 1 representing perfect replay fitness of the model to the event log, were used to assess the fit of the discovered process models (52). Baseline characteristics are presented as means and standard deviations (SD) for continuous variables and as frequency (percentage) for categorical variables. Contingency table analysis was conducted to explore differences in the proportion of adolescents involved in each event variable understudy (see Table 2) by mental health diagnostic category and calculate odds ratios (see Figure 1).

## 3. Results

Seven hundred and seventy-five adolescents (boys: 69%, *n* = 535; girls: 31%, *n* = 240) were included in the final case series. By age 19, over sixty percent of all adolescents (63%, *n* = 489) in the case series had a mental health related contact with a NSW Health service. This included a visit to a mental health outpatient service, a presentation at an emergency department, or a hospital admission where a mental health related diagnostic code was recorded. Before age 19, half of all adolescents in the case series (*n* = 390) had received a diagnosis for one of the mental health related categories included in this study (excluding “mental health diagnosis to be allocated” see Table 1), with almost a third of all adolescents receiving a diagnosis for more than one mental health category (31%, *n* = 238).

As shown in Table 1, receiving a stress or anxiety disorder diagnosis was the most common disorder amongst adolescents in the case series (26%, *n* = 200). This was followed by drug and alcohol related mental disorders (17%, *n* = 129) and mood disorders (16%, *n* = 123). Almost 20% of adolescents in the case series had attempted suicide or self-harmed (19%, *n* = 148). Adolescents were, on average, first diagnosed with impulse control disorders (M_age at first diagnosis_ = 13.7, SD = 2.86) or neurodevelopmental disorders (M_age at first diagnosis_ = 13.8, SD = 2.86) at a younger age than other disorders. Adolescents with a neurodevelopmental disorder diagnosis recorded the highest average number of NSW Health mental health related contacts (M _contacts _= 39, SD = 75.03).

### 3.1. Discovering childhood and adolescent developmental trajectories

To identify significant developmental trajectories for adolescent mental health problems, we modeled temporal correlations between time-ordered diagnoses and events.

#### 3.1.1. Trajectories to age 19

**Figure 2:**
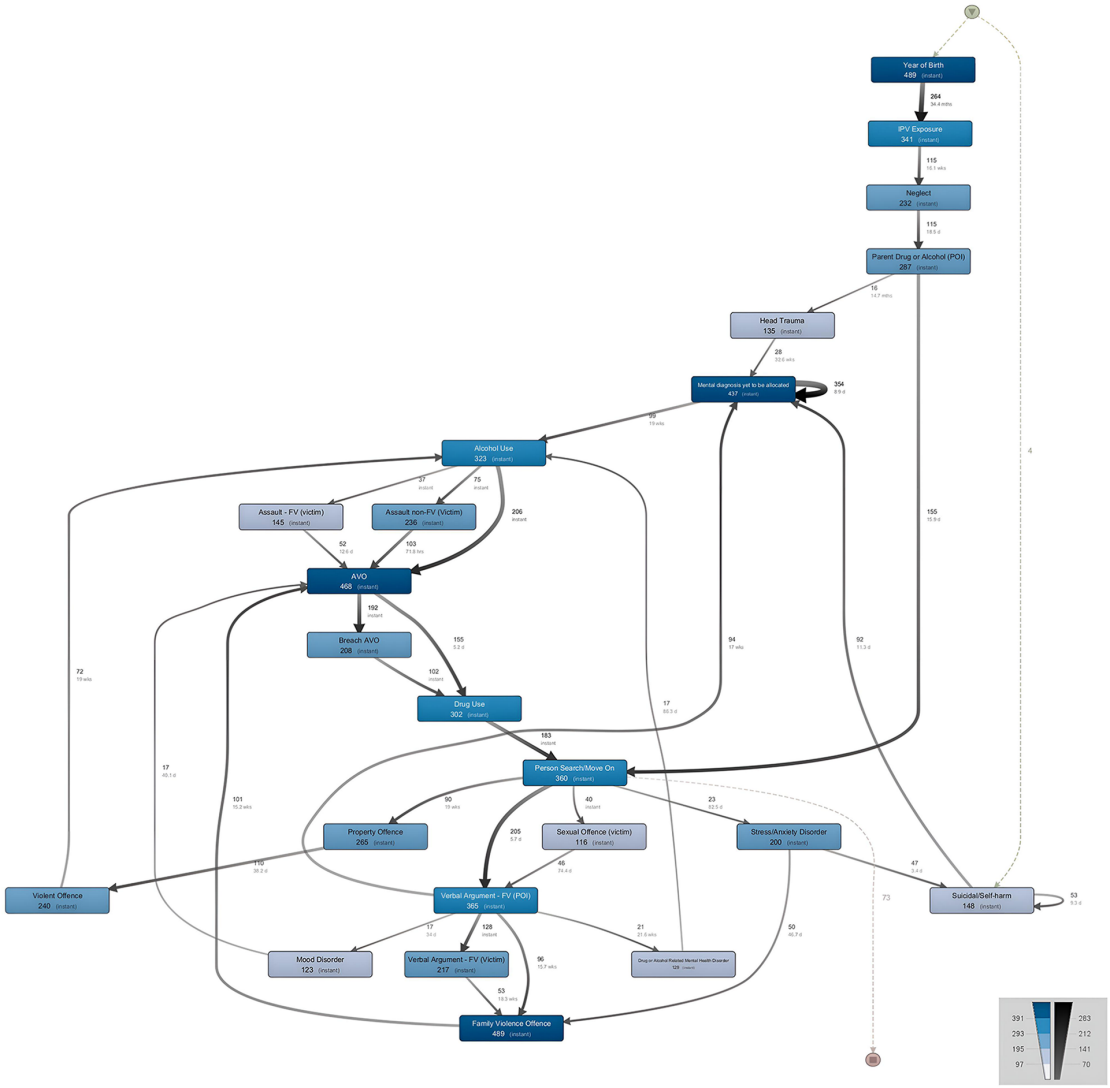
Process model from birth to age 19.

Initially, we modeled the temporal trajectories for diagnostic codes and events from birth to before age 19 for adolescents that had a recorded mental health related NSW Health contact (*n* = 489). The full process mining model had 23 nodes and 40 arcs. This model was filtered to include events with a minimum frequency of 15%, thus filtering out NSW Health contacts where a diagnosis for a neurodevelopmental disorder, impulse control disorder, psychotic disorder, personality disorder, or eating disorder was recorded due to the small number of adolescents receiving these diagnoses. The final model (see Figure 2) had a fitness of.78, suggesting that the model accurately represented 78% of the data as recorded in the event log.

In this model, the dominant primary events were parental intimate partner violence (*P*-IPV) exposure (70%, M_age at first exposure_ = 3.3 years, SD = 3.69), having a parent recorded as a person of interest in a NSWPF drug or alcohol related incident (53%, M_age of child at first parental involvement _= 4.8 years, SD = 5.06), and being recorded by the NSWPF as a child at risk of neglect or receiving a neglect related diagnosis (32%, M_age at first involvement_ = 10.5 years, SD = 4.67).

Almost 90% of adolescents (89%, *n* = 437) in the model had contact with a mental health outpatient service where “mental health diagnosis to be allocated” was recorded (M_age at first diagnosis_ = 14.3 years, SD = 2.34). For most adolescents in the case series this event was recorded before receiving a diagnosis for a mental health related disorder. For just over 20% of adolescents (21%, *n* = 92), involvement in a “mental health diagnosis to be allocated” event occurred an average of 11 days following an attempt suicide or self-harm event.

As seen in Figure 2, dominant events in the pathway from a “mental health diagnosis to be allocated” event to receiving a mental health disorder diagnosis involved drug (62%, M_age at first involvement_ = 15.2 years, SD = 1.92) and/or alcohol use (66%, M_age at first involvement_ = 15.0 years, SD = 1.83), involvement in a NSWPF person search or move on incident (74%, M_age at first involvement_ = 14.2 years, SD = 1.95), or being recorded as a person of interest in a family violence related verbal argument incident (75%, M_age at first involvement_ = 14.2 years, SD = 2.09). Eleven percent (*n* = 55) of all adolescents received a diagnosis for a drug or alcohol related mental health disorder (*n* = 21), a mood disorder (*n* = 17) or were involved in a “mental health diagnosis to be allocated” event (*n* = 17) directly after being involved as a person of interest in a family violence related verbal argument incident. These adolescents, on average, received these diagnoses within six months after the verbal argument incident. Almost a quarter of adolescents (24%, *n* = 47) with a stress or anxiety disorder diagnosis attempted suicide or self-harmed within an average of 3.4 days following a stress or anxiety disorder related diagnostic event. Over a third of all adolescents who attempted suicide or self-harm were involved in a second attempt suicide or self-harm incident within an average of nine days.

#### 3.1.2. Associations between events and mental health diagnosis categories

As seen in Table 2, the proportion of adolescents involved in each event variable was found to vary across mental health diagnostic categories. Compared to adolescents with other mental health disorders, proportionately more adolescents with a neurodevelopmental disorder were exposed to P-IPV, whereas proportionately more adolescents with a drug or alcohol related mental health disorder were recorded for having a parent involved in a drug and/or alcohol related police incident, neglect, or had a head trauma diagnosis.

As process mining analysis is limited in its capacity to investigate predictive associations between variables, odds ratios were calculated to examine the association between adolescents' involvement in each of the non-mental health related event variables (from birth to before age 19) and receiving a diagnosis for a mental health disorder. Odds ratios were calculated for each of the main mental health diagnostic categories displayed in Table 2. An association was deemed significant if the confidence intervals did not cross one (53). As seen in Figure 1, being recorded for neglect was associated with increased odds of having a diagnosis for a stress or anxiety disorder (OR = 1.41, 95% CI = 1.02–1.95), or drug or alcohol related mental health disorder (OR = 1.85, 95% CI = 1.26–2.70). Adolescents recorded as a victim of a sexual offense were almost 2.5 times more likely to have a diagnosis for a stress or anxiety disorder (OR = 2.48, 95% CI = 1.66–3.69) or had attempted suicide or self-harmed (OR = 2.33, 95% CI = 1.52–3.58) and almost two times more likely to be diagnosed with a mood disorder (OR = 1.77, 95% CI = 1.10–2.82), neurodevelopmental disorder (OR = 1.92, 95% CI = 1.67–3.17) or impulse control disorder (OR = 1.81, 95% CI = 1.07–3.05) compared to adolescents who were not the victim of a sexual offense. Being recorded as the victim of a non-family violence assault (OR = 1.60, 95% CI = 1.14–2.23) or family violence related verbal argument incident (OR = 1.48, 95% CI = 1.05–2.09) was also associated with increased odds of a stress or anxiety disorder diagnosis.

Different types of offending behaviors were found to be associated with different mental health categories. Adolescents involved in family violence verbal argument incidents, as a person of interest, were 1.4 to 1.8 times more likely to have a diagnosis for a stress or anxiety disorder (OR = 1.39, 95% CI = 1.01–1.93), drug or alcohol related mental health disorder (OR = 1.75, 95% CI = 1.19–2.57), or a mood disorder (OR = 1.64, 95% CI = 1.11–2.42) compared to adolescents not involved in family violence verbal arguments. Increased odds of having a neurodevelopmental disorder (OR = 1.68, 95% CI = 1.06–2.68) or drug or alcohol related mental health disorder (OR = 1.63, 95% CI = 1.07–2.48) were found amongst adolescents involved in violent offending (physical assault offenses).

## 4. Discussion

Employing data held by police and health services for a case series of adolescents from birth to 19 years, we transformed episodic diagnostic and event data into longitudinal person-based records. Discovery process mining was then used to map longitudinal process pathways, along with the time intervals between events in the pathways, towards adolescents receiving a mental health related diagnosis. The trajectories modeled in the current study confirm that adolescents in the case-series were not exposed to piecemeal or singular risks. Instead, their pathway to mental health problems was characterized by the clustering of inter-related adverse childhood events, adverse police involvement, and poly-victimization. A strength of the process-mining approach utilized is that the temporal patterns of diagnostic events are preserved. This is a significant improvement over earlier methodologies used to derive disease sequences (54). In addition, the long-time span of the data allowed us to evidence the more common co-morbidities and co-occurring events in the trajectories for childhood and adolescent mental health problems. To our knowledge, this study is the first to employ process mining to identify such childhood developmental trajectories.

The constellation of adversity experienced by adolescents in the current study confirms P-IPV and parental drug and alcohol use patterned with neglect, being the victim of a sexual offense, or the victim of a family violence incident, were dominant events in the pathway towards a mental health related diagnosis. Our findings align with earlier research that found higher rates of domestic violence in families of children with behavioral or emotional disorders (58%) or anxiety disorders (49%) and higher rates of substance use in families of children with mood (31%) or anxiety disorders (25%) (55). However, the study conducted by Naughton, Maybery (55) could not ascertain the temporal ordering of these events. Our study found that, on average, adolescents with a mental health diagnosis were first exposed to P-IPV or parental drug and/or alcohol use before the age of 5 and neglect before age 11. These findings suggest that the bundling of cumulative risk in childhood placed adolescents in the case series at a higher risk of poor mental health outcomes. In our sample, 70% (*n* = 341) of children who later went on to develop mental health related problems and 69% (*n* = 102) of those diagnosed with later self-harm or attempted suicide were exposed to *P*-IPV and/or parental drug and alcohol use in childhood. These early childhood parental characteristics, derived from administrative data, could be used for identifying at-risk children and families and targeting early-childhood mental health prevention or mitigation.

The current study found that involvement in events that increased the odds of adolescents having a diagnosis for a mental health disorder varied across mental health categories. Being the victim of a non-family violence related assault incident, family violence verbal argument incident, or neglect, all significantly increased the odds of adolescents having a stress or anxiety disorder, whereas being recorded as a child-at-risk of physical abuse increased the odds of being involved in an attempt suicide or self-harm incident. Consistent with previous research (56), being the victim of a sexual offense was found to significantly increase the odds of adolescents having a diagnosis for a stress or anxiety disorder, mood disorder, or attempt suicide or self-harm. In our study, being the victim of a sexual offense also increased the odds a having a diagnosis for a neurodevelopmental disorder or an impulse control disorder. Regardless of diagnosis type, prior research has found that a history of reported or unreported sexual abuse is common among people who have contact with mental health services (57). In our study, adolescents were, on average, first recorded as a victim of a sexual offense at age 11, occurring on average two to three years before adolescents' first mental health service contact. These findings confirm the importance of providing early and ongoing intervention efforts with children and adolescents who are victims of a sexual offense and other co-occurring childhood adversity to improve mental health outcomes.

From a preventative intervention perspective, our findings highlight the importance of responding earlier to mental health concerns, including the need for mental health support and services to be made more easily accessible for children and adolescents. The current study found a substantial increase in the number of adolescents (*n* = 209) receiving a mental health related diagnosis between the time of their first adolescent-to-parent family violence offense and age 19 years. A recent Australian study found that many adolescents involved in family violence offending were not linked to mental health services until after receiving a legal response (9). Our study found that involvement as a person of interest in a family violence verbal argument was a dominant event in the pathway to receiving a mental health diagnosis. This initial contact with police represents an opportunity for early family violence related intervention, including an opportunity for referral to adolescent mental health services.

Current responses to adolescent-to-parent family violence, including involvement in NSWPF reported verbal arguments, include the use of AVOs. Most of the adolescents in the present study (96%, *n* = 468) were issued a family violence related AVO. The use of AVOs as a response to adolescent-to-parent family violence has been questioned by researchers in the field (9, 58). In the current study breaching a family violence related AVO was associated with increased odds of having a diagnosis for all of the main mental health categories (with having a neurodevelopmental disorder being the exception). These findings suggest that when an adolescent is found to have a pre-existing mental health condition, assessments to ensure the adolescent can understand and comply with sentence outcomes or AVO conditions may be required (9).

This study has some limitations. The sample used for this study was a cohort of adolescents issued a legal action for an adolescent-to-parent violence offense. As incidents of family violence are known to be underreported to the police, the case series may only represent the most severe cases (59). Our analysis highlights segments of the population who are high users of secondary and tertiary mental health services, as well as repeat juvenile justice offenders. Compared to adolescents state-wide across NSW (per 1,000 population), rates of victimization (neglect: case series 334, NSW 21; sexual offense victimization: case series 196, NSW 26; family violence assault victimization: case series 261, NSW 16) and other offending (violent offending: case series 404, NSW 15; property offending: case series 493, NSW 23) were higher amongst adolescents in the case series compared to the general population (60). Rates of mental health issues were also higher in the case series compared to the rate per 1,000 population nationwide across Australia (stress or anxiety disorders: case series 258, Australia 88) (61). This resonates with earlier data linkage research (62) that demonstrated individuals with higher rates of criminal court attendance also tended to be higher users of other government services such as welfare and accidental injury insurance (63) and, as such, may not represent the general population of adolescents who have a mental health diagnosis. Finally, there may be other factors that contribute to the described trajectories that were not collected in this study, such as child protective services involvement, parental attachment, parental health and mental health, parental criminal history, socioeconomic status, housing stability, level of school engagement, and peer associations. The influence of these factors in the trajectory toward mental health issues warrants attention in future research.

This study provides valuable insights into the developmental trajectories of a cohort of at-risk young people. The significant chronicity of childhood adversity observed in this cohort highlights that early identification of at-risk children must be a continued focus of child health services in order to reduce and identify early emerging mental health problems.

## Data Availability

The datasets presented in this article are not readily available because of ethical restrictions surrounding the public release of this dataset.
